# Surgical Technique and Clinical Analysis of Twelve Cases of Isolated Laparoscopic Resection of the Hepatic Caudate Lobe

**DOI:** 10.1155/2018/5848309

**Published:** 2018-01-16

**Authors:** Bin Jin, Zhengchen Jiang, Sanyuan Hu, Gang Du, Binyao Shi, Du Kong, Jinhuan Yang

**Affiliations:** Qilu Hospital of Shandong University, Jinan, Shandong 250012, China

## Abstract

**Objective:**

To describe the surgical procedures of laparoscopic caudate lobectomy and analyze its clinical efficiency for treating cancer.

**Methods:**

Twelve consecutive patients of hepatocellular carcinoma, hepatic hemangioma, and focal nodular hyperplasia who received laparoscopic caudate lobectomy in Qilu Hospital of Shandong University from January 2013 to January 2017 were included in this study. The clinical data, intraoperative parameters, and postoperative outcomes were assessed.

**Results:**

All 12 patients received totally laparoscopic technique. The operative time was 140.8 ± 95.34 minutes. The average estimated blood loss was 97.92 ± 90.54 ml, and no blood transfusions were required. The mean duration of hospital stay was 9.17 ± 2.88 days. There was no perioperative complication or patient mortality in this series.

**Conclusions:**

Laparoscopic caudate lobectomy is safe and feasible in the selected patients.

## 1. Introduction

Laparoscopic surgery, as an important part for the minimally invasive surgery, has been extensively used in clinical practices nowadays. In 1991, Reich et al. initially describes their technique on laparoscopic hepatectomy in three women [1]. Two years later, Wayand and Woisetschiager reported their experiences on removal of a solitary liver metastasis by laparoscopic technique in a 63-year old patient [2]. In China mainland, laparoscopic hepatectomy was firstly performed in 1994 [3]. Afterwards, such technique has been commonly used in our country.

The scope of liver resection ranged from benign lesions to hemihepatectomy, difficult segmental resection, and even hepatectomy for liver transplantation. In the past decades, caudate lobectomy was considered as a challenge as the caudate lobe of liver was deep in site and was adjacent to the inferior vena cava (IVC), portal vein, and hepatic veins [4, 5]. Nowadays, laparoscopic caudate lobectomy is no longer a restricted zone with the advances of the medical technique [6, 7]. However, there are still some difficulties in the procedures especially the hemorrhage. In this study, we reported our experiences on 12 cases who underwent laparoscopic caudate lobectomy.

## 2. Materials and Methods

### 2.1. Patients

Twelve patients admitted to our department from January 2013 to January 2017 were included in this study. Among the 12 cases, 4 (33.33%) were diagnosed with cavernous hemangioma, 7 (58.33%) with primary liver cancer, and 1 (8.33%) with focal nodular hyperplasia in liver. All cases showed no ascites. The liver function was classified as Child-Pugh A class. Each patient signed the informed consent. The study protocols were approved by the Ethics Committee of Qilu Hospital of Shandong University.

### 2.2. Surgical Procedures

**Figure 1:**
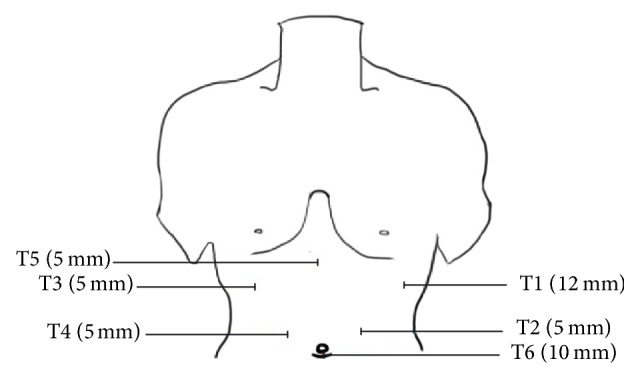
Port position for laparoscopic isolated caudate lobectomy.

The patients were placed in a supine position (30 degrees anti-Trendelenburg). Pneumoperitoneum was established through a subumbilical port with a pressure of 12 mmHg. Then six-port technique was used (Figure 1, T1–T6). The Trocar and laparoscope were inserted.

**Figure 2:**
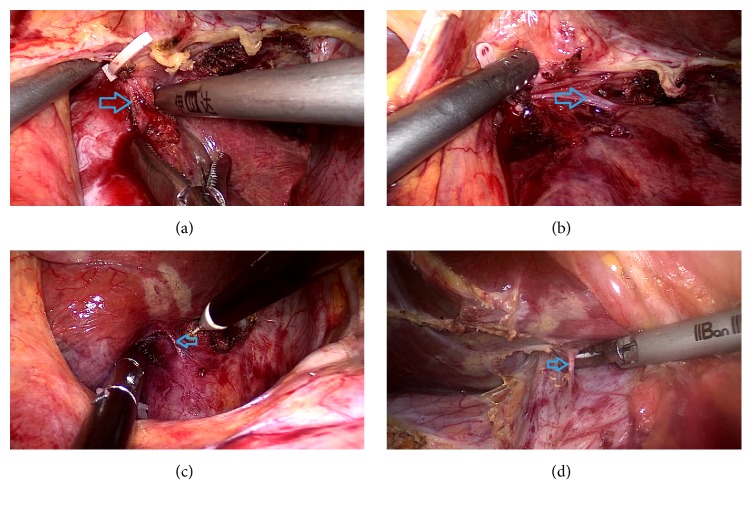
Transection of the CL branches from the IVC or porta hepatis. (a),(b) Dissection of SHV. (c), (d) Dissection of Glissonian branches of the CL.

**Figure 3:**
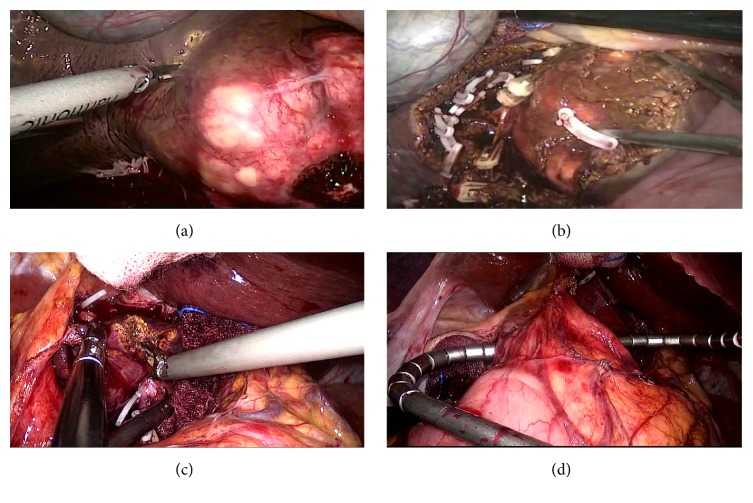
Transection of the CL parenchyma and technique of exposure to surgical vision. (a)–(c) Dissection of left or right of caudate lobe. (d) Place the S-shaped retractor to pull the hepatoduodenal ligament.

For the short hepatic vein, we firstly open the lesser omentum to expose the tumor in the caudate lobe. Then the third porta of liver was dissected, and then the short hepatic veins in conjunction with the caudate lobe were dissected. Subsequently, the short hepatic veins were clamped using a Hem-o-lok (Figures 2(c) and 2(d)). For the management of the first and second porta hepatis, the vessels in conjunction with the caudate lobe including hepatic artery and portal vein were dissected and then clamped using the Hem-o-lok clamp and the absorbable clamp (Figures 2(a) and 2(b)). When separating the hepatic pedicle, the assistant can pull caudate lobe downwards to expose the Glissonian branches. The chief surgeon can ligate the branches one by one. This method reduced the risk of vascular injury and avoided the incidences of postoperative complications. Subsequently, an S-shaped retractor was used to pull the first porta hepatis to the right side (Figure 3(d)). Then the caudate lobe was transected using a combination of ultrasonic dissection from the left side. The large vessels and bile duct were clamped using a Hem-o-lok, absorbable clamp, and titanium clamp. The first porta hepatis was pulled to the left side. Liver tissues at the right side of the tumor in the caudate lobe were treated using a similar method (Figures 3(a)–3(c)). During the operation, special cares were taken to protect the IVC, the portal vein, and the bile duct. Then an intraperitoneal drainage tube was inserted. Finally, the specimen was placed in an endocatch bag and extracted through the subumbilical port.

## 3. Results

### 3.1. Patient Characteristics

Among the 12 cases, 7 (58.3%) showed hepatic cirrhosis and 7 (58.3%) showed chronic hepatitis B (Table 1). The surgery duration was 140.8 ± 95.34 min. The intraoperative blood loss was 97.92 ± 90.54 ml. The mean hospitalization duration was 9.17 ± 2.88 days. No postoperative hemorrhage, liver failure, infection, or mortality was noticed. All the patients were followed up for a duration of 12–15 months. No recurrence was noticed in these patients.

Postoperative recovery of liver function showed that alanine aminotransferase (ALT) and aspartate aminotransferase (AST) were higher in all patients compared with the baseline levels. Despite ALT and AST showed decrease on postoperative days 3 and 5, the levels were still higher than the baseline levels. This may be related to the injury of the adjacent liver tissues in the process of operation or ischemia-reperfusion injury caused by blocking the porta hepatis (Table 2).

## 4. Discussion

There are still some disputes about the scope for the caudate lobe. In 1955, Couinaud divided the liver into eight segments (S1–S8) according to the distribution pattern of the intrahepatic vessels, among which caudate lobe was defined as S1. In 1985, Kumon divided the caudate lobe into three sections, including the Spiegel lobe, the paracaval portion, and the caudate process [27]. The Spiegel lobe was located in a position behind the lesser omentum, to the left of the Arantius ligament. In addition, the paracaval portion that was attached to the anterior IVC surface through retrohepatic ligament and short hepatic veins was localized at the right side of the Spiegel lobe. As the smallest of the three sections, caudate process is a thin tongue-like projection that was localized between the IVC and the portal vein to the right of the paracaval section. The upper border of the caudate lobe extended to a position that was behind the major hepatic veins. To the best of our knowledge, it is still a challenge for the exposure of caudate lobe as it was adjacent to IVC, portal vein, and hepatic vein, which induces much blood loss or complications and high mortality rate after open surgery.

In 1987, Philipmonet et al. firstly performed laparoscopic cholecystectomy. Later, the technique is extensively used in clinical practice as it shows exciting characteristics such as minimally invasive, less blood loss, and slight injury. As the largest substantial organ in human body, liver has abundant blood supply such as proper hepatic artery and portal vein. Besides, its anatomical structure was rather complex and the hemostasis was difficult in clinical practice, which hampered the application of laparoscopic technique. In recent years, with the development of microsurgery, laparoscopic hepatectomy has been widely used in clinics [28–31]; however, laparoscopic caudate lobectomy is still involving harsh technical demanding. In the past decades, due to technical limitations, the majority of laparoscopic caudate lobectomy is mainly restricted to the anterior hepatic segment (S2–S6). In 2006, Dulucq et al. reported their experiences on laparoscopic approach for caudate lobectomy in 2 cases [32]. After a literature review until July 2017, we have only found 67 cases who underwent laparoscopic caudate lobectomy, and most of the studies are presented as case reports (Tables 3 and 4) [8–23, 25, 26, 32]. In this study, we reported our experience on laparoscopic hepatic caudate lobectomy. The surgery was all successfully performed, with no cases transferred to the laparotomy. The surgical duration was 140.8 ± 95.34 min, and the intraoperative blood loss was 97.92 ± 90.54 ml. The total hospitalization duration was 9.17 ± 2.88 days. After the surgery, no hemorrhage, liver failure, infection, or death was noticed. Based on our experiences, we suggested that surgeons should pay attention to the following aspects in clinical practice.

For the selection of laparoscopic approach, it should be based on the lesion site, size, and the liver function of the patients. In general, the left-sided laparoscopic approach is suitable for the Spiegel lobe and the patients with a tumor diameter of <3 cm. The right-sided laparoscopic approach is mainly suitable for the paracaval lesions and the caudate process. The anterior approach and the combination between the left- and right-sided laparoscopic approaches are suggested for the cases with the whole caudate lobe involvement. Compared with the combination between the left- and right-sided laparoscopic approaches, the anterior approach is more suitable for the patients with large size lesions. Asahara et al. [24] proposed that patients with a lesion diameter of >4 cm could receive anterior approach. Peng et al. [33] suggested anterior approach for the cases with massive lesions or involvement of inferior vena cava and short hepatic vines, as it could prevent the hepatic rotation and hepatic venous rupture. Thus, it is the best choice for the caudate lobectomy. On this basis, the combination of the left- and right-sided laparoscopic approaches is more suitable after taking the limitation of the telescope devices as it could help to free the caudate lobe in the tumor site, which prevents the vascular injuries.

Hemostasis and exposure of surgical field are crucial for those received caudate lobectomy. Vascular inflow to CL is derived from primary Glissonian branches originating from the right and left portal veins. Meanwhile, the hilar bifurcation branch mainly contributes to the supply of the paracaval portion and Spiegel lobe to the left portal veins and the caudate process on the right, which is convenient for blocking vessels. Studies have shown that the Glissonian branches of the CL are short and unfocused, forming a pedicle form when entering the CL. Therefore, when dealing with it, it should be close to the CL [12]. CL commonly has two arteries with one contributing to the blood supply of the paracaval portion and Spiegel lobe originating from left hepatic artery or middle left hepatic artery, while the other one contributes to the blood supply of the caudate process originating from right hepatic artery. Other studies have shown that there is a confluence between the arteries of the CL and the arteries around the bile duct, which indicated that hepatic portal occlusion should be selected for the caudate lobectomy. In this study, a self-designed tube was used for the hepatic portal occlusion via the first porta hepatis, in order to block the blood supply in the porta hepatis in an intermittent manner. Such method induced less blood loss and prevented liver ischemia-reperfusion injury.

The venous vein of the CL is usually conformed into the IVC in the form of the SHV, which is featured by thin vascular wall, short trunk, and a deep location [34]. There are usually two to four SHV injected into the left and right side of the IVC. The thick SHV is usually localized at the middle and lower parts of the CL; however, no SHV is noticed in the upper part. Therefore, a safe passage can be established between the bilateral SHV and the superior right hepatic artery [10, 12].

The argon knife can be used for the hemostasis at the liver surface. Compared with the previous techniques, the argon knife contributes to quick hemostasis and less tissue injury, which then reduces the surgical duration and possibility of postoperative hemorrhage [35]. Meanwhile, argon knife showed satisfactory killing effects on the resident cancer cells. For those received laparoscopic caudate lobectomy, the procedures promoted the successful rate in the presence of a limited surgical field. In cases of poor hemostasis using argon knife, some options should be selected such as vascular clamp and suturing.

Liver is an important source for the synthesis of coagulation factors. When liver function was impaired, the coagulation factor synthesis was downregulated, which then resulted in decreased scavenging of the tissue thromboplastin and the activated fibrinolytic factors, as well as prothrombin time (PT) and INR. Some patients showed higher PT and INR after surgery compared with the baseline level, indicating that the operation had some damage to liver function. Meanwhile, the operation showed less damage to coagulation function. In some patients, bilirubin showed an upward trend on days 1 and 3 compared with the baseline levels, which may be related to the partial liver tissue injury. Fortunately, the bilirubin level decreased on day 5. Most of the postoperative patients showed a decrease in albumin on days 1 and 3, which may be related to the surgical trauma and the nutritional status of postoperative patients. However, the degree of reduction is not significant, indicating that the operation had less damage to albumin synthesis.

In summary, laparoscopic caudate lobectomy contributed to the attenuation of wound and decrease in hospitalized duration. Based on our experiences, laparoscopic caudate lobectomy is safe in clinical practice after understanding its indications and presence of complete presurgical preparation.

## Figures and Tables

**Table 1:** Results of seven patients undergoing laparoscopic caudate lobectomy.

Patient no.	Age/gender	HBV	Cirrhosis	Other diseases	Resection	Tumor origin	Tumor size (mm)	Operation time (min)	EBL (ml)	HPCT (min)	POS (days)	Drainage time (days)	Conversion
(1)	29/M	−	−		CL	HCH	62*∗*52	85	50	15	7	0	-
(2)	23/F	−	−	Chronic gastritis	CL	FNH	51*∗*35	205	350		6	4	-
(3)	59/F	+	+	Coronary disease	CL	HCC	52*∗*53	140	100		7	5	-
(4)	50/F	−	−		CL	HCH	51*∗*55	115	100	21	10	5	-
(5)	58/M	+	+		CL	HCC	61*∗*43	150	200	20	15	13	-
(6)	47/F	−	−	Hypertension	CL	HCH	52*∗*35	75	50		8	4	-
(7)	48/M	+	−	Renal clear cell carcinoma	CL	HCC	65*∗*54	420	50		14	12	-
(8)	50/M	+	+		CL	HCC	52*∗*50	130	60		8	5	-
(9)	54/F	+	−		CL	HCC	49*∗*48	90	50		9	4	-
(10)	49/F	−	−		CL	HCH	51*∗*55	100	55		12	6	-
(11)	60/F	+	+		CL	HCC	54*∗*53	80	60		8	5	-
(12)	51/M	+	+		CL	HCC	61*∗*56	100	50		6	5	-

CL: caudate lobe; PMT, HPCT: hepatic pedicle clamping time; HCH: hepatic hemangioma; HCC: hepatocellular carcinoma; EBL: estimated blood loss; POS: postoperative hospital stay; HCA: hepatocellular adenoma.

**Table 2:** Perioperative data and follow-up outcomes.

No.	Presurgery						Postoperative day 1						Postoperative day 3				Postoperative day 5			
	ALT	AST	TBIL	ALB	PT	INR	ALT	AST	TBIL	ALB	PT	INR	ALT	AST	TBIL	ALB	ALT	AST	TBIL	ALB
(1)	14	17	8.4	42.2	12	1.14	126	62	21.1	42.9	13.7	1.3	60	40	18	42.8	23	26	14.8	42.8
(2)	6	12	10.6	44.4	11.1	1.06	89	76	89	76	12.3	1.15	59	27	7.1	42	29	17	7.4	40.9
(3)	14	22	10.5	42.8	11.9	1.13	90	110	11	35.7	13.3	1.26	69	55	14.9	41.1	38	24	14.2	42.7
(4)	15	20	9.9	45	12.4	1.18	90	72	13.5	42.2	12.1	1.15	44	24	11.4	39.7	27	17	10.5	41
(5)	13	19	10.1	44.3	12	1.14	53	73	16.8	36.1	13.8	1.32	23	24	26.1	34.7	13	11	21.3	38.4
(6)	17	21	8.3	45.1	11.6	1.10	50	45	9.7	39	12.4	1.17	27	17	11.3	40.2	38	27	7.9	41.6
(7)	20	21	13.9	42.9	11.1	1.06	86	75	20.1	42.8	12.6	1.2	50	30	24.6	39.9	29	26	10.6	37
(8)	17	18	10.3	45.2	11.2	1.13	73	82	19.5	42.3	12.3	1.15	48	34	15.6	43.5	25	14	12.5	47
(9)	10	15	8.5	43.5	12	1.11	81	75	17.4	41.5	12.6	1.14	60	29	14.7	42.9	19	18	11.8	45.3
(10)	9	20	11.0	44.9	11.5	1.09	65	69	16.4	43.9	11.9	1.14	35	37	13.2	44.2	24	23	10.3	45.3
(11)	13	19	9.3	43.7	12.2	1.12	68	66	15.2	43.2	12.5	1.19	59	36	14.1	43.9	25	19	10.5	44.1
(12)	15	21	8.9	44.0	11.5	1.08	72	70	16.5	42.9	12.1	1.15	48	40	15.2	44.1	21	17	9.8	45

ALT: alanine aminotransferase (U/L); AST: aspartate transaminase (U/L); TBIL: total bilirubin (umol/L); ALB: albumin (g/L); PT: prothrombin time (s); INR: international normalized ratio.

**Table 3:** Reported cases of laparoscopic isolated caudate lobectomy.

Patient no.	First author	Year	Case	Tumor origin	Tumor size (mm)	Operation time (min)	EBL (ml)	POS (day)
(1)	Araki [8]	2006	2	Metastases (2)		105–150	100–200	8, 10
(2)	Cheung [9]	2007	1	HCH	52	160	50	8
(3)	Inamori [10]	2007	7	Benign (5), HCC (2)				
(4)	Lai [11]	2011	4	HCH (3), hepatolithiasis (1)				
(5)	Kyriakides [12]	2012	3			180–300	150–400	
(6)	Jin [13]	2012	1	HCA		77	50	
(7)	Cai [14]	2013	1	Benign	60			
(8)	Gringeri [15]	2014	7	HCC (4), metastases (3)	45	210–345	10–1000	
(9)	Zarzavadjian Le Bian [16]	2014	2	HCC	15	137, 150	137, 150	4, 5
(10)	Salloum [17]	2014	1	HCA	60	270	200	
(11)	Kokkalera [18]	2014	1	Metastases		140	80	
(12)	Ho [19]	2015	2	Metastases		140	80	6, 8
(13)	Oh [20]	2016	5	Metastases (4), FNH (1)	35	240 (180–345)	200 (50–700)	6–14
(14)	Yoon [21]	2016	6	HCC (1), CCC (1), metastases (1)	2.65 (0.9–5.1)	382 (168–615)	242.5 (120–360)	7 (6–13)
(15)	Jiang [22]	2016	15	Metastases (12), HCC (1), benign (2)	50	150 (60–480)	75 (0–500)	8 ± 6.5
(16)	Chen [23]	2016	1	HCC	45	190	90	
(17)	Asahara [24]	2016	1	HCC	16	270	270	
(18)	Ishizawa [25]	2016	1	Metastases	20	180	220	
(19)	Koffron [26]	2016	4	HCC (1), Metastases (1), HCH (2)	11.8 (1.2–20)	185 (155–350)	250 (200–600)	7 (4–11)

HCA: hepatocellular adenoma; HCH: hepatic hemangioma; HCC: hepatocellular carcinoma; EBL: estimated blood loss; POS: postoperative hospital stay.

**Table 4:** Reported cases of laparoscopic isolated caudate lobectomy.

No	First author	Mean op time (min)	Mean EBL (ml)
1	Araki [8]	127.5	150
2	Cheung [9]	160	50
6	Jin [13]	77	50
9	Zarzavadjian Le Bian [16]	143.5	143.5
10	Gringeri [15]	270	200
11	Kokkalera [18]	140	80
12	Ho [19]	140	80
13	Oh [20]	240	200
14	Yoon [21]	382	242.5
15	Jiang [22]	150	75
16	Chen [23]	190	90
17	Asahara [24]	270	270
18	Ishizawa [25]	180	220
19	Koffron [26]	185	250

Other reports (*n* = 14)		189.64 ± 77.77	150.07 ± 79.38
This study (*n* = 10)		140.8 ± 95.34	97.92 ± 90.54
		*P* = 0.163	*P* = 0.131

HCA: hepatocellular adenoma; CCC: cholangiocarcinoma; HCH: hepatic hemangioma; HCC: hepatocellular carcinoma; EBL: estimated blood loss.
